# Establishing a Relationship between In Vitro Potency in Cell-Based Assays and Clinical Efficacious Concentrations for Approved GLP-1 Receptor Agonists

**DOI:** 10.3390/pharmaceutics16101310

**Published:** 2024-10-08

**Authors:** Alessandro Boianelli, Pär Nordell, Joseph Earl, Jacqueline Naylor, David Hornigold, Rasmus Jansson Löfmark, Monika Sundqvist

**Affiliations:** 1 DMPK, Research and Early Development Cardiovascular, Renal and Metabolism (CVRM), BioPharmaceuticals R&D, AstraZeneca, 431 83 Mölndal, Sweden; par.nordell@astrazeneca.com (P.N.); rasmus.jansson.lofmark@astrazeneca.com (R.J.L.); monika.sundqvist@astrazeneca.com (M.S.); 2 Bioscience Metabolism, Research and Early Development, Cardiovascular, Renal and Metabolism (CVRM), BioPharmaceuticals R&D, AstraZeneca, Cambridge CB21 6GH, UK; joseph.earl@astrazeneca.com (J.E.); david.hornigold@astrazeneca.com (D.H.); 3 Bioscience Metabolism, Research and Early Development, Cardiovascular, Renal and Metabolism (CVRM), BioPharmaceuticals R&D, AstraZeneca, 431 83 Mölndal, Sweden; jacqueline.naylor@astrazeneca.com

**Keywords:** GLP-1 agonists, in vitro in vivo correlation, PK/PD modelling, model informed drug development (MIDD)

## Abstract

**Background:** Glucagon-like peptide-1 receptor agonists (GLP-1RAs) play an important role in the treatment of type 2 diabetes (T2D) and obesity. The relationship between efficacy and dosing regimen has been studied extensively for this class of molecules. However, a comprehensive analysis of the translation of in vitro data to in vivo efficacious exposure is still lacking. **Methods:** We collected clinical pharmacokinetics for five approved GLP-1RAs to enable the simulation of exposure profiles and compared published clinical efficacy endpoints (HbA1c and body weight) with in-house in vitro potency values generated in different cell-based assays. Additionally, we investigated the correlation with target coverage, expressed as a ratio between the steady state drug exposure and unbound potency, body weight, or HbA1c reduction in patients with T2D. **Results:** We found that the best correlation with in vivo efficacy was seen for in vitro potency data generated in cellular assays performed in the absence of any serum albumin or using ovalbumin. Residual variability was larger using in vitro potency data generated in endogenous cell lines or in the presence of human serum albumin. For the human receptor assay with no albumin, exposures above 100-fold in vitro EC50 resulted in >1.5% point HbA1c reduction, while a 5% BW reduction was related to approximately 3× higher exposures. A similar relationship was seen in the ovalbumin assay. **Conclusions:** Overall, the relationship established for in vitro potency and in vivo efficacy will help to increase confidence in human dose prediction and trial design for new GLP-1RAs in the discovery and early clinical phases.

## 1. Introduction

Glucagon-like peptide-1 receptor agonists (GLP-1RAs) act on the Gs-coupled G protein-coupled receptor (GPCR) glucagon-like peptide-1 receptor (GLP-1R) to stimulate glucose-dependent insulin release from the pancreatic islets, reduce food intake, and delay gastric emptying [1,2]. GLP-1RAs have been used to treat T2DM for almost two decades and have more recently been approved for the treatment of obesity since 2015 (reviewed in Popoviciu et al. 2023 [3,4]). Currently, there is also a significant clinical development focusing on dual and triple agonist molecules that can activate the GLP-1R plus one or more other targets (reviewed in Camilleri and Acosta 2023 [5,6]). The most reported side effects for GLP-1RAs are gastrointestinal and include nausea, vomiting, and diarrhea [7,8]. To reduce adverse events, most GLP-1RAs are titrated in the clinic, whereby dose levels are increased over time to slowly adapt the body to the efficacious exposure.

Endogenous GLP-1 is rapidly inactivated by dipeptidyl peptidase-IV (DPP-IV) and has a half-life of just 2–3 min. Therefore, the development of a viable therapeutic GLP-1 analog with increased metabolic stability and extended half-life has been imperative. GLP-1RAs can now be divided into short-acting (exenatide twice daily, lixisenatide), intermediate (liraglutide), and long-acting (albiglutide, dulaglutide, semaglutide, and exenatide once weekly (QW)) [4,9]. Exenatide was the first GLP-1RA approved and is based on the structure of the Gila monster exendin-4 peptide, which, unlike native human GLP-1, includes amino acid modifications that confer resistance to DPP-IV-mediated degradation [10]. Lixisenatide has a similar structure to exenatide but with an additional six c-terminal lysines to further improve stability [11]. Half-lives for these short-acting GLP-1 agonists are around 2–3 h. Liraglutide is a human GLP-1 analog with a 16C fatty-acid conjugation via a glutamic acid spacer in position 26 that confers binding to human serum albumin, leading to an increased half-life into the 13 h range. This albumin binding could be further improved in semaglutide with a C18 di-acid linked by a γGlu-2xOEG linker to the same position as for liraglutide [12] resulting in a half-life of around 5 days. Alternate approaches to extend half-life were used for albiglutide, which consists of a GLP-1 dimer fused to recombinant human albumin [13], and dulaglutide, a DPP-IV-protected GLP-1 analog covalently linked to a human IgG4-Fc heavy chain by a peptide linker [14]. Exenatide QW has achieved a prolonged exposure profile by formulating the compound in microspheres to slowly release free drugs over time into the systemic circulation. Semaglutide, albiglutide, dulaglutide, and exenatide QW can all be delivered once weekly [4,9,14,15]. Semaglutide is the only GLP-1RA currently available both in a subcutaneous and an oral formulation, with the oral tablet being approved in 2019 [16], but currently, there are several GLP-1RAs in the clinical phase aiming for oral delivery [17,18].

During the development of new compounds, in vitro screening plays a major role in ranking and benchmarking molecules; however, a further complexity is that the setup for GLP-1 receptor in vitro assays can vary significantly, leading to vastly different estimates of potency. These differences can arise from both assay system set-up and analysis [19]. Within the same host cell system, agonist potency will vary depending on receptor expression level, potential receptor reserve, and coupling efficiency when comparing between cell lines [20,21,22]. Furthermore, for evaluating lipidated and non-lipidated peptides, the method used for sample preparation and the choice of non-specific blocking (NSB) reagent to prevent binding to plasticware must be considered [23,24]. For the preclinical characterization of GLP1-RA molecules, several different NSB reagents and concentrations have been employed across different research groups, including 0.1% bovine serum albumin (BSA) [25], 0.1% ovalbumin (OA) [24], 0.1% bovine Casein [26,27], or no NSB [28]. Despite NSB reagents preventing the binding of peptides such as GLP1-RAs to plasticware and, therefore, improving potency [23,24], they can lead to different estimates of potency for the same GLP-1RAs between different assay formats due to the differential binding of the GLP-1RAs to proteins. Moreover, understanding plasma protein binding for lipidated peptides in a physiologically relevant level of serum albumin is key to understanding half-life improvement.

**Figure 1:**
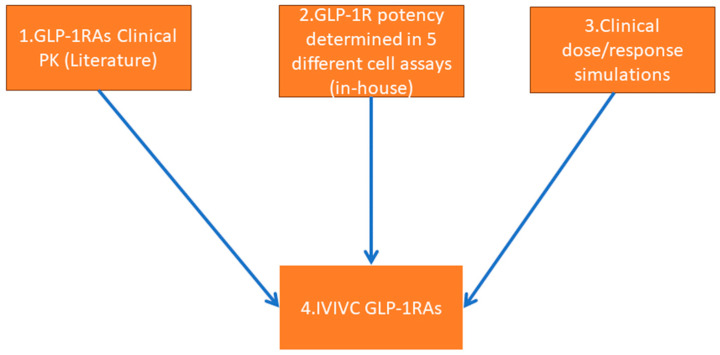
Graphical representation of the workflow. **1. Collection of Clinical Pharmacokinetics (PK):** Data on the clinical pharmacokinetics of five GLP-1 receptor agonists (GLP-1RAs) was collected from existing literature. **2. In vitro Determination of GLP-1R Potency:** The potency of GLP-1 receptor agonists in vitro was assessed using five different assay setups. **3. Simulation of Clinical Dose**–**Response Information:** Information on clinical dose–response in a relevant disease population was simulated from comparediabetesdrugs.com. **4. Evaluation of In vitro to in Vivo Potency correlation (IVIVC):** Consistency in therapeutically relevant clinical exposure in relation to in vitro potency was analyzed to identify which assay format provides the most predictive human pharmacokinetic-pharmacodynamic (PK/PD) relationship.

A recent publication presented a thorough evaluation of dose–response relationships [29] for approved antihyperglycemic drugs, utilizing an extensive model-based meta-analysis [30]. At the time of submission, the database encompassed over 300 studies on more than 30 drugs belonging to 6 pharmacological classes. Furthermore, this platform allows for the generation of treatment dose-effect simulations https://www.comparediabetesdrugs.com (accessed on 10 January 2024). However, due to the significant heterogeneity in the structure, protein binding, potency, dosing regimen, and pharmacokinetics of these drugs, the dose–response information cannot be directly utilized for predicting the efficacy of pre-clinical candidates. In this work, we have established the relationship between (1) clinical exposures, (2) in vitro potency generated in well-characterized GLP-1R expressing cell lines [25], and (3) simulated efficacy endpoints (body weight and HbA1c) for clinically approved GLP-1R mono agonists (Figure 1). This facilitates predictions of efficacious concentrations in humans for new drug entities and evaluation of the (4) predictive power of the in vitro assay set-ups.

## 2. Materials and Methods

### 2.1. In Vitro GLP-1 Receptor Agonist Potency

Cell-based cyclic adenosine monophosphate (cAMP) accumulation assays were used to measure in vitro potency for GLP-1RAs in a stable Chinese hamster ovary (CHO) cell line expressing human GLP-1R (generated at AstraZeneca) or EndoC-βH1 cells endogenously expressing the GLP-1R (Endocells, Paris, France) as previously described [25]. In brief, test GLP-1RA serial dilutions were prepared using an ECHO^®^ acoustic liquid handler (Beckman, High Wycombe, UK) to obtain an 11-point dose–response curve in assay buffer (Hanks’ balanced salt solution (HBSS)) supplemented with 25 mM of HEPES and 0.5 mM of 3-isobutyl-1-methylxanthine (IBMX). Buffers were supplemented with bovine serum albumin (BSA, Sigma-Aldrich, Milwaukee, WI, USA), ovalbumin (OA, Sigma-Aldrich, Milwaukee, WI, USA), or human serum albumin (HSA, Sigma-Aldrich, Milwaukee, WI, USA) at reported concentrations to minimize NSB or to replicate in vivo plasma protein binding at a physiologically relevant concentration of human serum albumin (4.4%). The EndoC-βH1 cell assay was performed in an assay buffer containing 0.1% BSA.

Following compound and cell incubation for 30 min, the level of cAMP accumulation was determined using a cAMP dynamic 2 HTRF (homogeneous time-resolved fluorescence) kit (Cisbio, Codolet, France), following the two-step protocol according to the manufacturer’s guidelines. Treated cells were incubated with anti-cAMP cryptate and cAMP-d2 in lysis buffer at room temperature for 1 h and read on an Envision plate reader (PerkinElmer, Waltham, MA, USA). Data was converted to %Delta F as per the manufacturer’s guidelines, and the results were analyzed via a 4-Parameter Logistical Analysis with samples graphed as % activation plots with assay window defined by negative control as basal cell cAMP levels and positive control defined by native GLP-1 agonist signal.

### 2.2. Human Plasma Protein Binding

Measurements of plasma protein binding are essential to understand the free drug exposure. Lipidated peptides have variable plasma protein binding characteristics. Non-lipidated GLP-1RAs dulaglutide and exenatide have been shown not to bind to human plasma proteins; therefore, estimating EC_50_ in vitro at different protein concentrations does not lead to changes in the estimation (Table S1). For semaglutide, liraglutide, and lixisenatide, values from the literature were used [31,32,33]. The values used are shown in Table S2.

### 2.3. Clinical Pharmacokinetics

Human pharmacokinetic (PK) data or PK parameters were collected from the literature for the five different GLP-1RAs: liraglutide, semaglutide (oral and subcutaneous), dulaglutide, lixisenatide, and exenatide [BID and QW] (Table S3) [34,35,36,37,38,39]. In cases where random effects models were available, the population PK parameters were used. The compounds exhibited linear PK in the relevant range except for dulaglutide, which had a slight non-linear bioavailability reported [34]. Maximum concentration (C_max_), area under the curve over a dosing interval (AUC_last_), average concentration (C_avg_ derived as AUC_0-Tau_/Tau), and trough concentration (C_min_) were estimated at steady state for different dose levels and are listed in Table S4.

### 2.4. HbA1c and Body Weight Simulations

Clinical main endpoints for GLP1-RAs (HbA1C and body weight) at therapeutic dose regimens were estimated for a representative disease population using the Clinical Trial Simulator available at https://www.comparediabetesdrugs.com (accessed on 10 January 2024). Importantly, this advanced analysis tool appropriately adjusts for significant confounding factors such as the duration of treatment, baseline levels, background treatment (drug-naive or metformin), and titration schedules. In short, the change from baseline in HbA1c and body weight were simulated at defined dose regimens for evaluated GLP-1R mono agonists (exenatide BID [Byetta] 20 ug s.c. BID; exenatide QW [Bydureon] 1, 2, 3 mg s.c. QW; dulaglutide 0.25, 0.5, 0.75, 1, 1.25, 1.5, 1.75, 2, 2.5, 3, 4 mg s.c. QW, lixisenatide 10, 20, 30 s.c. QD, liraglutide 0.3, 0.6, 0.9, 1.2, 1.8, 2.5 mg s.c. QD; semaglutide QW [Ozempic] 0.25, 0.5, 0.75, 1, 1.25, 1.5, s.c. QW, semaglutide QD [Wegovy] 1, 2, 3, 5, 7, 8, 10, 12, 14, 17, 20 mg oral QD), against a placebo group for a 50% drug naive population with 50% of subjects on metformin treatment. Baseline values for HbA1c and body weight were set to 8.5% and 100 kg, reflecting the typical trial population characteristics. Median HbA1c and body weight reduction with an associated 90% credible range by week 52 of treatment were calculated for each drug and dose regimen collected from 100 virtual trials using a fixed sample size of 100 subjects (100 × 100 simulated trials).

### 2.5. Exposure Normalisation

Model estimates of C_avg_, C_max_, and C_min_ at the steady state for each GLP-1RA and regimen were converted to a normalized EC_50_ (nEC_50_) multiple for each drug and regimen as follows: Firstly, EC_50_ values associated with each in vitro assay format (Table 1) was normalized by the assay-specific EC_50_ measured for endogenous GLP-1 (7-36) NH_2_ peptide (EC_50,GLP-1_).

(1) $$nEC50=\frac{{EC}_{50,drug}}{{EC50}_{GLP1}},$$

Secondly, the clinical exposures in relation to nEC_50_, considering the expected free fraction in the in vitro and in vivo setting (free: free), were calculated for each assay format:

(2) $$fold\;nEC50\left(0.1\%\;BSA\right)=\frac{C_{P}{\;\times \;f}_{unb,p}}{{nEC}_{50}\left(0.1\%\;BSA\right)\times f_{unb}\left(0.1\%\;SA\right)},$$

(3) $$fold\;{nEC}_{50}\left(0\%\;SA\right)=\frac{C_{P}\times f_{unb,p}}{n{EC}_{50}\left(0\%\;SA\right)},$$

(4) $$fold\;{nEC}_{50}\left(0.1\%\;OA\right)=\frac{C_{P}{\;\times \;f}_{unb,p}}{{nEC}_{50}\left(0.1\%\;OA\right)},$$

(5) $$fold\;{nEC}_{50}\left(0.1\%\;HSA\right)=\frac{C_{P}\times f_{unb,p}}{{nEC}_{50}\left(0.1\%\;HSA\right)f_{unb}\left(0.1\%\;SA\right)},$$

(6) $$fold\;{nEC}_{50}\left(4.4\%\;HSA\right)=\frac{C_{P}}{n{EC}_{50}\left(4.4\%\;HSA\right)},$$

where C_P_ are either the average (C_avg_), max (C_max_), or trough (C_trough_) plasma concentrations at the steady state, f_unb,p_ is the unbound fraction in plasma, and f_unb_ (0.1% SA) is the free fraction in diluted serum albumin (SA) conditions, estimated from

(7) $$f_{\mathrm{unb}}\left(0.1\%\;SA\right)=\frac{{DF\times f}_{\mathrm{unb},p}}{1+\left(DF-1\right)\times f_{\mathrm{unb},p}},$$

assuming a dilution factor (DF) of 4.4%/0.1% = 44. Calculated values are listed in Table S4.

### 2.6. Regression Analysis and PKPD Modelling Strategy

A sequential analysis procedure was set up with three main purposes based on the given response (BW and Hb1Ac) simulations, clinical PK, and in vitro potency data: (i) order the relevance of derived in vitro potency from selected assay formats, (ii) assess whether C_avg_, C_max_, or C_min_ as a PK exposure metric in relation to in vitro potency and in relation to clinical outcomes, and (iii) define where acceptable correlation is observed the most descriptive PKPD model by which early predictions of the efficacy of novel GLP-1 mono agonists can be generated in absence of clinical data.

The initial stage included a model-independent assessment of the degree of association between potency ratio and treatment effect by Spearman correlation analysis using the *cor.test* function included in the R *stats* package (R Statistical Software v4.0.0; R Core Team 2021). Rank-ordering, with regards to assay format and PK metric, was based on the strength of the relationship as determined by the correlation coefficient r (+/− 1 indicates perfect correlation and 0 indicates no association) and the *p*-value reflecting the probability that the true r = 0. A correlation coefficient > 0.75 for either of the endpoints and a *p*-value < 0.01 was set as inclusion criteria for subsequent model-based regression analysis evaluating three different direct response models: a linear (y = a · x), a power (y = a · x^b^), and a sigmoidal (y = a + (b − a) · x^c^/(x^c^ + d^c^)) drug effect (x represents fold nEC_50_ from different assays, y represents BW or HbA1C change and a to d represent model parameters) using MATLAB 2020b *fit* function (The MathWorks Inc., Natick, MA, USA). Residuals were weighted by the number of dose levels to balance the impact of simulated data for each drug and evaluated based on parameter identifiability as measured by the associated confidence range and goodness-of-fit measures, including the corrected AIC.

## 3. Results

### 3.1. In Vitro GLP-1 Receptor Agonist Potency

**Figure 2:**
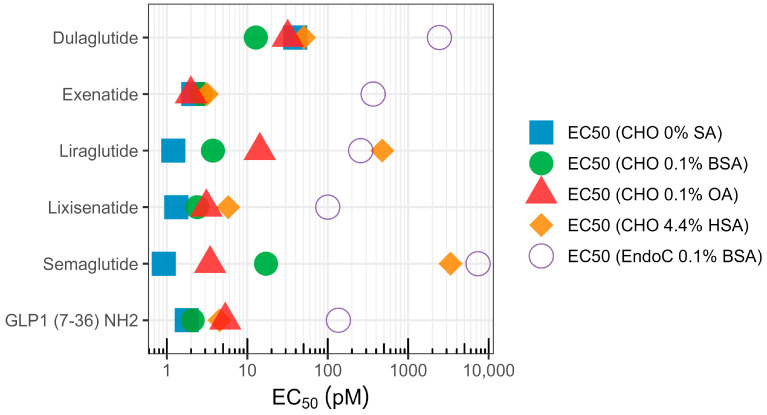
Comparison of in vitro potency (EC_50_) determined using a stable Chinese hamster ovary (CHO) cell line expressing human GLP-1R or EndoC-βH1 cells endogenously expressing the GLP-1R at specified serum albumin conditions (BSA = bovine serum albumin, OA = ovalbumin, HSA = human serum albumin). Table 1 provides the numeric values and associated standard deviations.

In vitro potency data is summarized in Table 1 and Figure 2. The potency of the reference peptide GLP-1(7-36)NH2 was in the single-digit pM range in all overexpressing receptor CHO assay formats but reduced in the EndoC-based assay where GLP-1 has a lower potency, as predicted from lower endogenous receptor expression levels previously demonstrated in Kenakin et al., 2017 [20]. The compounds with a low/non-existent protein binding (exenatide, dulaglutide) had relatively similar potencies in all CHO-based assay formats while being less potent in the EndoC assay. The highly protein-bound compounds (liraglutide, semaglutide) exhibited less potency with increased protein concentration in the assay, as expected. Semaglutide (a stearate acylated GLP-1 analog) showed a lower EC_50_ value in OVA 0.1% compared to BSA 0.1%. However, liraglutide (palmitate acylated GLP-1 analog) showed a lower EC_50_ value in BSA 0.1% compared to OVA 0.1%

### 3.2. Clinical Pharmacokinetics

**Figure 3:**
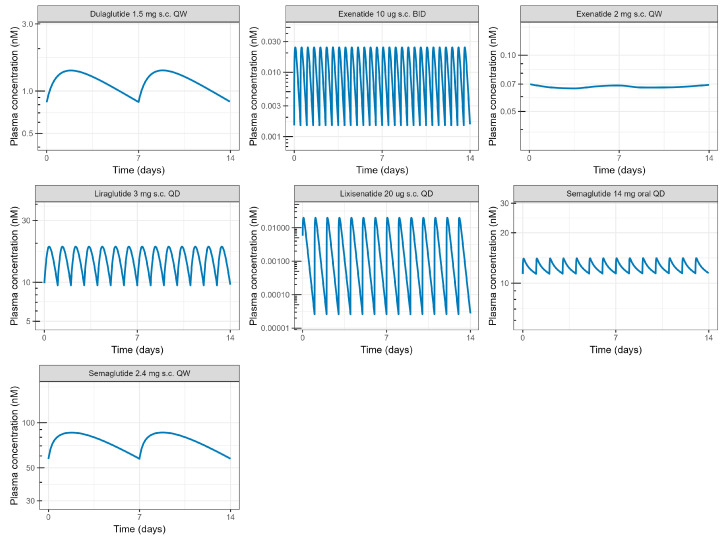
Model predicted steady state plasma PK at selected dose regimens for different GLP-1As. Estimates of C_avg_, C_max_, and C_trough_ used in the PK/PD analysis were extracted from generated profiles.

Estimates of the representative clinical steady state of the C_avg_, C_max_, and C_min_ at relevant dose levels (Table S3) were derived from simulated profiles using published PK model parameters as listed in Table S3. A 14-day exposure simulation at the steady state for the relevant therapeutic dose level for the different GLP-1RA is shown in Figure 3. The greatest peak-to-trough ratio (C_max_/C_min_) was estimated for lixisenatide (>500), which displays an almost complete washout over the 24 h dose interval, followed by exenatide BID (19). Ratios for other drugs were estimated to be ≤2.

### 3.3. PK/PD Analysis

**Figure 4:**
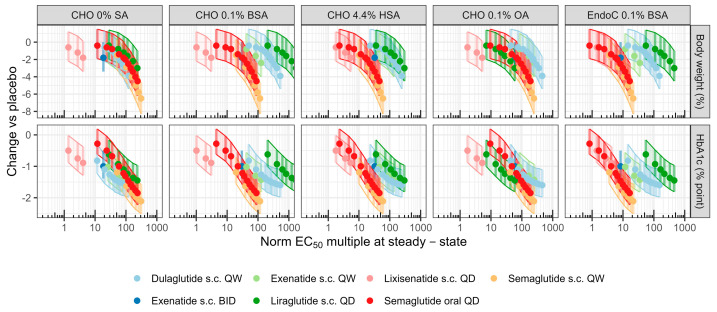
Simulated bodyweight (%, top) and HbA1c (% point, bottom) change from placebo (comparediabetesdrugs.com; 100 × 100 trials over 52 weeks; median with 90% credible range indicated) for the average fold normalized GLP-1R EC_50_ at the steady state (Methods). Panels left to right show multiples derived using the evaluated cell systems (CHO, EndoC) serum albumin conditions (0%, 0.1% BSA, 0.1% OA, 4.4% HSA). Treatment effects vs. multiples for C_max_ and C_trough_ are given in Supplementary Information.

Simulated treatment effects (bodyweight and HbA1c) relative to the placebo associated with each drug and dose regimen were plotted against derived clinical PK exposure metrics at the steady state (C_avg_, C_max_, or C_min_) divided by measured in vitro GLP-1 normalized potency (nEC50) after correction for albumin binding (Figure 4). The resulting PK/PD relations for folds over unbound EC_50_ for each in vitro potency assay are shown in Figure 3, while corresponding relations based on C_max_ and C_min_ are given in Figures S1 and S2, respectively. The highest treatment effect among included drugs and doses resulted from semaglutide 1.5 mg s.c. QW, simulating a 6.5% reduction in body weight (4.7–8.3%) and a 2.1%-point reduction in HbA1c (1.6–2.5% point) in this patient population. Across drugs, body weight reduction was right-shifted compared to HbA1c, lowering as expected from published dose–response analysis [29].

### 3.4. Regression Analysis

**Figure 5:**
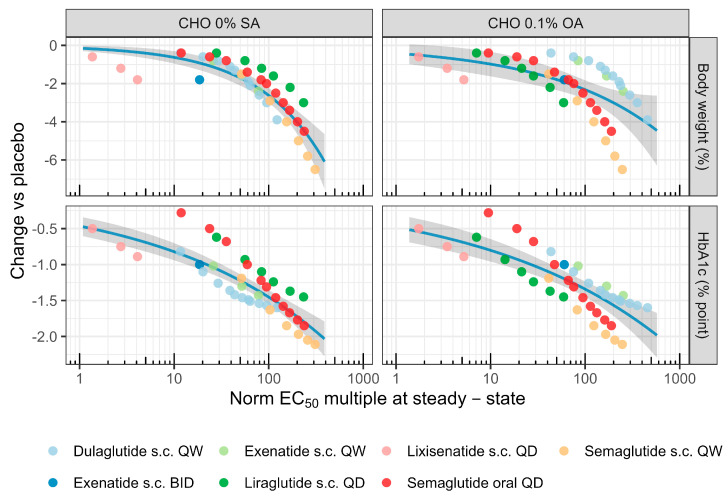
Power model fit on PK/PD data for body weight and HBA1c placebo corrected reduction for CHO_0%SA_ and CHO_0.1%OA_ assays. Blue line represents median fit, while gray shaded area represents 5–95% confidence interval fit.

Correlation coefficients (r) and associated *p*-values based on a Spearman analysis applying either C_avg_, C_max_, or C_min_ as exposure metrics are listed in Table S5. Generally, multiples of nEC_50s_ from CHO_0.1%BSA_, CHO_4.4%HSA_, and EndoC_0.1%BSA_ displayed a low to moderate negative correlation to weight and HbA1c (−0.17 > r > −0.51), whereas a significantly stronger Spearman correlation with potency from the CHO _0%SA_ and CHO _0.1%OA_ assays were observed. Coefficients based on C_avg_ as the PK metric were −0.87/−0.85 for body weight/HbA1c with potency in CHO 0% SA and −0.81/−0.66 for the same endpoints in CHO 0.1% OA (all *p*-values < 10^−5^). Using either C_max_ or C_min_ did not significantly improve rank-ordering, and hence, C_avg_ was used as a proxy for the effect driving concentration in the subsequent analysis, applying potency either from 0% SA or 0.1% OA conditions. Next, the appropriateness of increasingly complex PK/PD models to quantitatively describe the relations was evaluated. Table S6 summarizes the model fit diagnostics for each drug effect function (linear, power, or sigmoidal), assay format (0% SA or 0.1% OA), and endpoint (body weight or HbA1c). Acceptable parameter precision, along with improved RMSE and AICc, were obtained with a power vs a linear model for both HbA1c and body weight at both conditions. While a further reduction in RMSE could be obtained with a sigmoid model, parameter precision was poor, indicating over-fitting, along with an increased AICc. Best-fit representations with 95% functional prediction bounds are shown in Figure 5 and Table 2. Based on the 0% SA assay, the model indicates that an average potency multiple of 114 [88–159] was associated with a 1.5% point reduction in HbA1c vs placebo and that a 5% BW reduction requires almost three-fold as high exposure (283 [228–418]). Prediction ranges based on the 0.1% OA assay were wider but overlapped those of the assay without albumin.

## 4. Discussion

During discovery and drug development, benchmarking new molecules against clinically approved compounds in preclinical assays is routine. In particular, predicting human doses using in vitro potency and predicted clinical PK helps rank compounds and assess projected dose acceptability. However, this approach becomes substantially less informative if there is limited data demonstrating that in vitro potency can be used to project therapeutic drug exposure [40]. GLP-1RA in vitro cell-based assays can report varying potencies depending on assay set-up (cell type, receptor expression level, protein concentration, etc.). In addition, clinically approved GLP-1RAs display very different kinetics and protein binding, further complicating translation. In this study, we aim to gain a deeper understanding of an in vitro assay format that is most predictive for the human PK/PD relationship and that can be used, together with predicted human PK, to support human dose prediction for new chemical entities.

We show that the predictivity of a GLP1-RA in vitro assay is highly variable and dependent on the assay format but, in the right setting, can predict human efficacy across modalities. The highest correlated assay (the CHO_0%SA_ assay) for our dataset indicates that an exposure approximately 100-fold over a normalized EC_50_ is required for a clinically meaningful reduction in HbA1c. Such a ratio is much higher than expected for a GPCR [39]. This is likely explained by a high receptor density in the in vitro assay setting, which results in greater potency than observed in vivo. A high receptor density giving rise to higher potency for GLP-1 assays has previously been shown in [19] and is also highlighted in the public assessment report of Mounjaro [41]. These results also indicate that a higher exposure is needed to reach significant body weight effects than is required to lower HbA1c. A body weight reduction of 5% or more likely requires exposure of above 300-fold nEC_50_ in our assays. The requirement for a higher exposure to drive body weight reduction is already established and is reflected in the approved doses for Ozempic versus Wegovy and Victoza versus Saxenda, although here we provide a quantitative and generalized assessment of the size of exposure increase required. The reason for the necessary increase in exposure between endpoints is not clear, but as GLP-1 receptors are present in several tissues in the body [41], the required exposure levels and required target engagement could differ for site and mechanism of action dependent on differences in receptor expression and receptor coupling [19,42]. In the current analysis, the focus has been on patients with diabetes, although body weight reductions are higher with GLP1-RAs in patients who are healthy obese.

Our analysis indicates that exposure over potency is the driver for higher body weight reduction and that higher doses would then result in greater effects. However, the frequency of nausea and vomiting is often the major cause for discontinuation of GLP1-RA drugs and may be a dose-limiting factor. We have focused on C_avg_ as the primary determinant of effect, as quantitatively assessing the most useful exposure estimate is challenging due to small peak-trough ratios for most compounds within the dataset. Any indication of differences is solely driven by exenatide BID and lixisenatide with peak-trough ratios above 10-fold. The trough concentrations for these two compounds do fall outside the range of the other compounds, which indicates that minimum concentrations are not the driving force behind GLP1-RA-mediated effects.

Although this is an empirical relationship, it further emphasizes the need to establish reliable associations for the assay used to characterize new drugs. Assumptions that the correlations are straightforward could result in significantly underestimating the required concentration for effect and, as such, the required dose. There is no reason to assume the model will not work across other molecules or modalities if the receptor binding mode and target turnover are similar. There are currently several small molecule agonists in different stages of clinical development that could be added to the model after approval. Recently, more complex descriptions of the glucose-insulin system have been published using quantitative systems pharmacology [42,43]. Food intake and body weight have been described using the Hall model [44,45] and gastric emptying in Voronova et al. [46]. Such models are useful for a full understanding of the dynamics of the system but take time to develop and validate, especially if the purpose is to describe the actions of multiple drugs. For the full integration of glucose and insulin homeostasis, Hba1c and body weight are not in place. Interestingly, in Bosch et al., by using a modified version of the Hall model, the authors estimated the in vitro to in vivo EC_50_ relationship for body weight reduction for liraglutide and semaglutide. Taking the substantial differences between the modeling approaches into account, the derived value from Bosch et al. was estimated in a similar range as indicated here. The current work focused on understanding the efficacious exposure of GLP-1 agonists in relation to in vitro potency under different assay conditions. In a typical drug discovery project, this type of information would also be complemented with pre-clinical in vivo PKPD data to further strengthen the projection of the clinical therapeutic exposure.

Cell-based in vitro potency assays have typically used low-level albumin supplements to prevent non-specific binding to plasticware and enhance assay performance and robustness. More recently, the use of lipidation to increase peptide half-life in vivo has led to a greater understanding of albumin’s impact on potency determination [23,25]. It is clear that protein binding has an impact on our assays, but that the CHO_0%SA_ assay gave the best correlation to effect was somewhat unexpected due to the known problem of nonspecific binding mentioned above. It is possible that the problem is low for the compounds included in this study but may be higher for other chemical entities, such as highly lipophilic small molecules, that may be very difficult to run in an assay totally devoid of protein. The 0.1% OA assay is intended to confer protection against the non-specific binding but also to have less binding to compounds in general. Indeed, this assay also showed very good correlation results and quite a similar exposure–response relationship as the CHO 0% SA assay, indicating that it can be a good tool for any series that is suspected to bind non-specifically to plastic.

One could expect that the EndoC-βH1 assay would have given a better result in our comparison as this is a β-cell model with endogenous levels of target expression. The results in this assay are, however, variable and similar to the CHO assay with 0.1% BSA. The protein binding in these assay formats is predicted based on human protein binding, and although liraglutide and semaglutide have relatively similar human albumin binding, despite different lengths of the fatty acid chain [47], such a prediction is relatively uncertain as BSA, OVA, and HSA are structurally different. Semaglutide (a stearate acylated GLP-1 analog) showed a lower EC_50_ value in OVA 0.1% compared to BSA 0.1%. However, liraglutide (aliphatic palmitate acylated GLP-1 analog) showed a lower EC_50_ value in BSA 0.1% compared to OVA 0.1%. A key difference is that the lipid moiety in semaglutide has a free carboxylic group, whereas liraglutide does not [43]. Differences in potency for GLP1-RAs bound to albumins may be due to different amino acid residues in the binding pocket. BSA may have more positively charged amino acids exposed in the binding pocket; therefore, negatively charged lipid acylated to semaglutide would have higher affinity to BSA compared to OVA due to stronger ionic bonding. As a result, there is greater availability of unbound GLP-1 analogs to occupy the GLP-1 receptors and elicit a greater response, resulting in a leftward shift of the dose–response curve and higher potency.

A caveat in this work is that we have not measured the direct protein binding of the compounds to ovalbumin, as it is not trivial to measure this for the highly bound lipoylated peptides. We have instead tested the compounds with varying concentrations of ovalbumin contained in the assay buffer. These experiments demonstrate that although protein binding may be less than with other proteins, there was still a shift in potency for liraglutide and semaglutide with increasing ovalbumin concentrations, indicating some level of protein binding. Further work needs to be done regarding protein binding to ovalbumin.

## 5. Conclusions

In this paper, we describe the relationship between exposure and endpoint response for several GLP-1 RAs corrected for in vitro potency. This allows for a more accurate prediction of the likely required exposure for new drugs with the same target and mode of action. We conclude that the in vitro assay best suited to support the translation to human efficacy should not include serum albumin. We also highlight the importance of developing such in vitro-in vivo understanding to inform the usefulness of the potency assay beyond the ranking of compounds. The data also suggests that using ovalbumin as a protein in the in vitro assay clearly improves in vitro-in vivo translation compared to more routinely used BSA.

## Figures and Tables

**Table 1:** In vitro potency (EC_50_) estimated from concentration–response (cAMP) data using a stable Chinese hamster ovary (CHO) cell line expressing human GLP-1R or EndoC-βH1 cells endogenously expressing the GLP-1R.

Drug	CHO 0% SA EC_50_ (pM) (s.d. ^1^)	CHO 0.1% BSA EC_50_ (pM) (s.d.)	CHO 0.1% OA EC_50_ (pM) (s.d.)	CHO 4.4% HSA EC_50_ (pM) (s.d.)	EndoC 0.1% BSA EC5_0_ (pM) (s.d.)
Dulaglutide	39.4 (15.3)	16 (11.7)	31.8 (9.10)	50.6 (13)	2440 (2100)
Semaglutide	0.915 (0.36)	31 (22.4)	3.45 (1.60)	3400 (2200)	7380 (3200)
Liraglutide	1.2 (0.55)	1.56 (2.22)	14.3 (5.90)	475 (329)	257 (150)
Exenatide	2.14 (0.64)	2.32 (0.68)	1.99 (1.00)	3.15 (1.83)	366 (180)
Lixisenatide	1.31 (0.04)	3.16 (0.39)	3.1 (2.54)	5.79 (0.74)	100 (N/A)
GLP-1 (7-36) NH2	1.77 (0.55)	3.09 (1.00)	5.32 (1.48)	4.53 (2.71)	136 (110)

^1^ s.d.: standard deviation; N/A: not available.

**Table 2:** Median and 95% functional prediction bounds at specified response values, associated with it of a power model to PKPD data.

Assay	Parameter	Change vs. Placebo	C_avg_/nEC_50_ at s.s.
CHO_0%SA_	Bodyweight (%)	−5	283 (161–276)
	HbA1c (%point)	−1.5	114 (88–159)
CHO_0.1%OA_	Bodyweight (%)	−5	780 (390–n.d. *)
	HbA1c (%point)	−1.5	165 (116–281)

* Upper bound not identifiable.

## Data Availability

The raw data supporting the conclusions of this article will be made available by the authors on request.

## Supplementary Materials

The following supporting information can be downloaded at: https://www.mdpi.com/article/10.3390/pharmaceutics16101310/s1, Figure S1: PK/PD based on trough plasma exposure; Figure S2: PK/PD based on maximum plasma exposure; Table S1: Shift assay data; Table S2: fu values for different GLP-1RAs; Table S3: Pharmacokinetic parameters for different GLP1-RAs; Table S4: C_avg_, C_max_ and C_min_ summary for all the different GLP-1RA dosing regimens; Table S5: Parameters associated with non-parametric correlation analysis (Spearman) for each endpoint and assay potency; Table S6: regression using a linear, power and sigmoid drug effect model to describe relation to weight and HbA1c effect.
