# Supplementary material for: Establishing a Relationship between In Vitro Potency in Cell-Based Assays and Clinical Efficacious Concentrations for Approved GLP-1 Receptor Agonists
Source: Pharmaceutics. 2024 Oct 8;16(10):1310. doi: 10.3390/pharmaceutics16101310 (PMC11510446; doi:10.3390/pharmaceutics16101310)
Supplement: Supplementary file 1 [file pharmaceutics-16-01310-s001.zip › GLP1_IVIVC supplementary v3_review.pdf]

# Supplementary Information

**Establishing a relationship between in vitro potency in cell-based assays and clinical efficacious concentrations for approved GLP-1 receptor agonists**

**Alessandro Boianelli[1,§*], Pär Nordell[1,§], Joseph Earl[2], Jacqueline Naylor[3], David Hornigold[2], Rasmus Jansson Löfmark[1] and Monika Sundqvist[1]**

[1] DMPK, Research and Early Development Cardiovascular, Renal and Metabolism, BioPharmaceuticals R&D, AstraZeneca, Gothenburg, Sweden

[2]Bioscience Metabolism, Research and Early Development, Cardiovascular, Renal and Metabolism (CVRM), BioPharmaceuticals R&D, AstraZeneca, Cambridge, United Kingdom

[3]Bioscience Metabolism, Research and Early Development, Cardiovascular, Renal and Metabolism (CVRM), BioPharmaceuticals R&D, AstraZeneca, Gothenburg, Sweden

***Correspondence: alessandro.boianelli@astrazeneca.com;

[§] These authors contributed equally to the work

# Figures

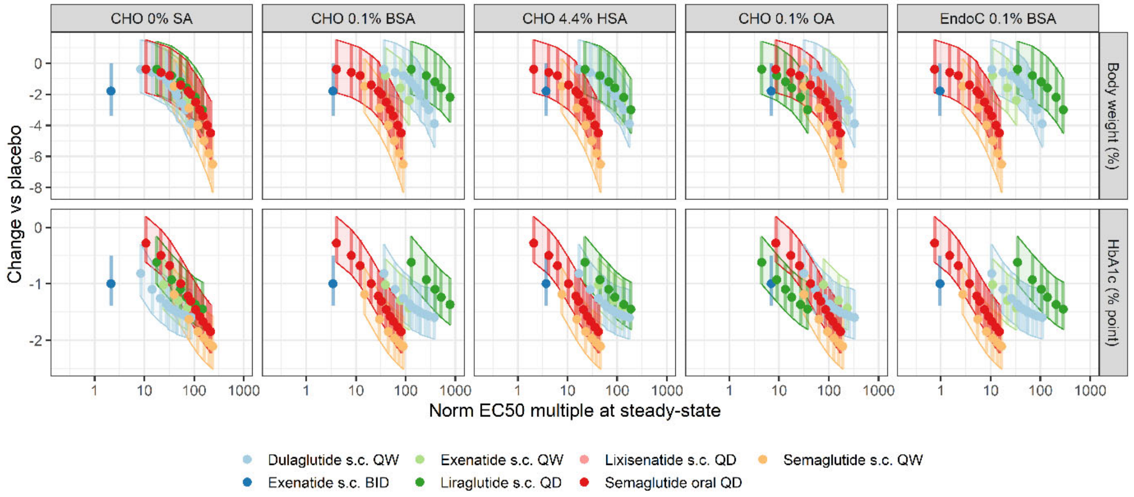


**Figure S1 PK/PD based on trough plasma exposure.** Simulated bodyweight (%, top) and HbA1c (% point, bottom) change from placebo (comparediabetesdrugs.com; 100 x 100 trials over 52 weeks; median with 90% credible range indicated) vs fold GLP-1R $EC_{50}$ at trough (Methods). Panels left to right shows multiples derived using the evaluated cell systems (CHO, EndoC) serum albumin conditions (0%, 0.1% BSA, 0.1% OA, 4.4% HSA).

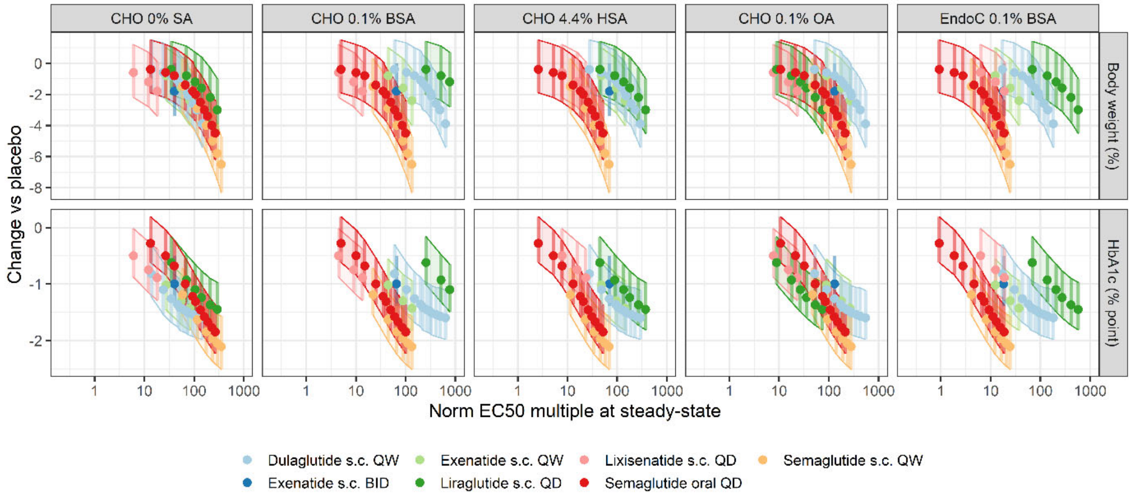


**Figure S2 PK/PD based on maximum plasma exposure.** Simulated bodyweight (%, top) and HbA1c (% point, bottom) change from placebo (comparediabetesdrugs.com; 100 x 100 trials over 52 weeks; median with 90% credible range indicated) vs fold GLP-1R $EC_{50}$ at $T_{max}$ (Methods). Panels left to right shows multiples derived using the evaluated cell systems (CHO, EndoC) serum albumin conditions (0%, 0.1% BSA, 0.1% OA, 4.4% HSA).

# Tables

**Table S1.** *In vitro* potency ($EC_{50}$) estimated from concentration-response (cAMP) data using a stable CHO cell line and incubated at 0.1, 0.3, 1 and 3% NSB where NSB = BSA (A) or OA (B).

A. NSB = BSA

| Compound | $EC_{50}$ (pM) 0.1% | $EC_{50}$ (pM) 0.3% | $EC_{50}$ (pM) 1% | $EC_{50}$ (pM) 3% |
|---|---|---|---|---|
| Dulaglutide | 9.07 | 10.8 | 12.1 | 9.7 |
| Semaglutide | 35 | 75.1 | 181 | 286 |
| Liraglutide | 3.9 | 7.59 | 18.2 | 58.1 |
| Exenatide | 2.7 | 1.68 | 1.5 | 1.58 |
| Lixisenatide | 2.77 | 2.72 | 4.25 | 2.25 |
| GLP1 (7-36 amide) | 2.14 | 2.03 | 3.91 | 5.11 |

B. NSB = OA

| Compound | EC50 (pM) 0.1% | $EC_{50}$ (pM) 0.3% | $EC_{50}$ (pM) 1% | $EC_{50}$ (pM) 3% |
|---|---|---|---|---|
| Dulaglutide | 46.4 | 34.1 | 30.7 | 26 |
| Semaglutide | 3.58 | 19.2 | 9.43 | 16.4 |
| Liraglutide | 26.9 | 33.8 | 136 | 285 |
| Exenatide | 3.19 | 2.62 | 3.75 | 2.33 |
| Lixisenatide | 6.25 | 2.69 | 3.44 | 3.46 |
| GLP1 (7-36 amide) | 5.44 | 4.05 | 8.34 | 10 |

**Table S2.** Estimated fraction unbound in human plasma $f_{unb,p}$ and 0.1% serum albumin $f_{unb}$ *(0.1% SA)*.

| Compound | $f_{unb,p}$ (%) | $f_{unb}$ (0.1% SA)[1] | Reference |
|---|---|---|---|
| Exenatide | 100 | 100 | Based on serum shift assay data in Table S1 |
| Dulaglutide | 100 | 100 | Based on serum shift assay data in Table S1 |
| Lixisenatide | 45 | 97 | [1] |
| Liraglutide | 0.51 | 18 | [2] |
| Semaglutide | 0.36 | 14 | [3] |

[1]Estimated from $f_{unb,p}$ (Methods).

**Table S3.** Pharmacokinetic parameters representing rate of absorption ($K_a$), central clearance ($CL$), central volume ($V_c$), peripheral volume ($V_p$), distribution clearance ($CL_d$), bioavailability ($F$) and lag-time ($T_{lag}$).

| Compound | $K_a$ ($h^{-1}$) | $CL$ (L/h) | $V_c$ (L) | $V_p$ (L) | $CL_d$ (L/h) | $F$ | $T_{lag}$ (h) | Population | Reference |
|---|---|---|---|---|---|---|---|---|---|
| Dulaglutide | 0.00769 | 0.0593 | 2.25 | 3.75 | 0.0201 | 0.47 at 1.5 mg | - | T2D | [4] |
| Semaglutide oral | 2.09 | 0.039 | 3.7 | 4.2 | 0.31 | 0.0069 | - | T2D,obesity | [5] |
| Semaglutide sc | 0.0253 | 0.039 | 3.7 | 4.2 | 0.31 | 0.847 | - | T2D | [6] |
| Liraglutide | 0.154 | 0.91 | 11.2 | - | - | 0.51 | 6 | T2D | [7] |
| Lixisenatide | 0.317 | 38.6* | 41.2* | | | | | T2D | [8] |
| Exenatide BID | 0.734 | 8.829* | 26.2* | | | | | T2D | [7] |
| Exenatide QW | Variable | 9.1* | 28.2* | | | | | T2D | [9] |

*CL/F and V/F

**Table S4.** Average ($C_{avg}$), maximum ($C_{max}$) and trough ($C_{min}$) exposure at steady-state.

| Compound | Exposure at steady-state | | |
|---|---|---|---|
| | $C_{max}$ (nM) | $C_{avg}$ (nM) | $C_{min}$ (nM) |
| Dulaglutide 1.5 mg s.c. QW | 1.4 | 1.2 | 0.83 |
| Exenatide 10 ug s.c. BID | 0.024 | 0.011 | 0.0015 |
| Exenatide 2 mg s.c. QW | 0.07 | 0.068 | 0.066 |
| Liraglutide 3 mg s.c. QD | 19 | 15 | 9.5 |
| Lixisenatide 20 ug s.c. QD | 0.02 | 0.0045 | 0.000026 |
| Semaglutide 14 mg oral QD | 14 | 12 | 11 |
| Semaglutide 2.4 mg s.c. QW | 86 | 75 | 57 |

**Table S5.** Parameters associated with non-parametric correlation analysis (Spearman) for each endpoint and assay potency. [1]A correlation coefficient rho of +/- 1 indicate perfect correlation and [2]the p-value reflects the probability that the true rho = 0.

| **PK metric** | $C_{avg}$ | | $C_{max}$ | | $C_{min}$ | |
|---|---|---|---|---|---|---|
| Spearman parameter | rho[1] | p value[2] | rho[1] | p value[2] | rho[1] | p value[2] |
| **CHO 0% SA** | | | | | | |
| HbA1c (% point) | −0.85 | $1.5 \times 10^{-12}$ | −0.85 | $1.7 \times 10^{-12}$ | −0.85 | $3.7 \times 10^{-12}$ |
| Body weight (%) | −0.87 | $1.0 \times 10^{-13}$ | −0.89 | $6.3 \times 10^{-15}$ | −0.88 | $6.5 \times 10^{-14}$ |
| **CHO 0.1% BSA** | | | | | | |
| HbA1c (% point) | −0.42 | $6.7 \times 10^{-3}$ | −0.40 | $9.3 \times 10^{-3}$ | −0.42 | $6.0 \times 10^{-3}$ |
| Body weight (%) | −0.27 | $9.2 \times 10^{-2}$ | −0.27 | $9.3 \times 10^{-2}$ | −0.27 | $8.8 \times 10^{-2}$ |
| **CHO 0.1% OA** | | | | | | |
| HbA1c (% point) | −0.81 | $1.1 \times 10^{-10}$ | −0.81 | $8.8 \times 10^{-11}$ | −0.82 | $7.7 \times 10^{-11}$ |
| Body weight (%) | −0.66 | $2.8 \times 10^{-6}$ | −0.68 | $1.0 \times 10^{-6}$ | −0.66 | $2.2 \times 10^{-6}$ |
| **EndoC 0.1% BSA** | | | | | | |
| HbA1c (% point) | −0.35 | $2.5 \times 10^{-2}$ | −0.26 | $1.0 \times 10^{-1}$ | −0.38 | $1.5 \times 10^{-2}$ |
| Body weight (%) | −0.20 | $2.0 \times 10^{-1}$ | −0.17 | $3.0 \times 10^{-1}$ | −0.21 | $1.8 \times 10^{-1}$ |
| **CHO 4.4% HSA** | | | | | | |
| HbA1c (% point) | −0.47 | $2.1 \times 10^{-3}$ | −0.42 | $5.7 \times 10^{-3}$ | −0.51 | $6.1 \times 10^{-4}$ |
| Body weight (%) | −0.34 | $2.8 \times 10^{-2}$ | −0.33 | $3.7 \times 10^{-2}$ | −0.37 | $1.7 \times 10^{-2}$ |

**Table S6.** Parameter point estimates with 90% confidence interval resulting from regression using a linear, power and sigmoid drug effect model to describe relation to weight and HbA1c effect. Linear model: y = a · x; Power model y = a · xb; Sigmoid model y = a + (b - a) · xc/(xc + dc). AICc = corrected Akaike information criterion, rmse = root mean square error. NaN = not a number, indicating ambigous fit obtained using specified model.

| Potency assay | Endpoint | Model | a | b | c | d | rmse | AICc |
|---|---|---|---|---|---|---|---|---|
| CHO 0% SA | Body weight | linear | -0.01 (-0.012 to -0.0085) | | | | 0.28 | -109 |
| | | power | -0.46 (-0.55 to -0.38) | 0.25 (0.21 to 0.29) | | | 0.086 | -205 |
| | | sigmoid | 0 (NaN to NaN) | -2.5 (NaN to NaN) | 0.45 (0.36 to 0.54) | 47 (36 to 58) | 0.094 | -187 |
| | HbA1c | linear | -0.021 (-0.024 to -0.019) | | | | 0.38 | -82.9 |
| | | power | -0.15 (-0.24 to -0.057) | 0.62 (0.5 to 0.75) | | | 0.33 | -94.9 |
| | | sigmoid | 0 (NaN to NaN) | -2.5 (NaN to NaN) | 0.94 (0.15 to 1.7) | 11 (-0.19 to 23) | 0.51 | -47.7 |
| CHO 0.1% OA | Body weight | linear | -0.0079 (-0.0092 to -0.0066) | | | | 0.28 | -106 |
| | | power | -0.48 (-0.6 to -0.36) | 0.22 (0.17 to 0.27) | | | 0.1 | -190 |
| | | sigmoid | 0 (NaN to NaN) | -2.5 (NaN to NaN) | 0.41 (0.32 to 0.51) | 64 (46 to 82) | 0.11 | -177 |
| | HbA1c | linear | -0.015 (-0.018 to -0.012) | | | | 0.58 | -47.7 |
| | | power | -0.42 (-0.74 to -0.087) | 0.37 (0.21 to 0.53) | | | 0.47 | -66.1 |
| | | sigmoid | 0 (NaN to NaN) | -2.5 (NaN to NaN) | 0.93 (0.19 to 1.7) | 11 (-1.2 to 24) | 0.52 | -46.5 |

# References


1. chmp, *Lyxumia*. 2012, EMA.
2. Ungewiss, J., S. Gericke, and H. Boriss, *Determination of the plasma protein binding of liraglutide using the escalate* equilibrium shift assay.* Journal of Pharmaceutical Sciences, 2019. **108**(3): p. 1309-1314.
3. CHMP, *Ozempic Assesment report*. 2017, EMA.
4. Geiser, J.S., et al., *Clinical pharmacokinetics of dulaglutide in patients with type 2 diabetes: analyses of data from clinical trials.* Clinical pharmacokinetics, 2016. **55**: p. 625-634.
5. Overgaard, R.V., et al., *Clinical pharmacokinetics of oral semaglutide: analyses of data from clinical pharmacology trials.* Clinical Pharmacokinetics, 2021. **60**: p. 1335-1348.
6. Overgaard, R.V., et al., *Population pharmacokinetics of semaglutide for type 2 diabetes.* Diabetes Therapy, 2019. **10**: p. 649-662.
7. Watson, E., et al., *Population pharmacokinetics of liraglutide, a once-daily human glucagon-like peptide-1 analog, in healthy volunteers and subjects with type 2 diabetes, and comparison to twice-daily exenatide.* J Clin Pharmacol, 2010. **50**(8): p. 886-94.
8. Frank, T., *Population pharmacokinetics of lixisenatide, a once-daily human glucagon-like peptide-1 receptor agonist, in healthy subjects and in patients with type 2 diabetes.* J Pharm Drug Deliv Res, 2013. **2**: p. 1.
9. Trägårdh, M., et al., *Input estimation for extended-release formulations exemplified with exenatide.* Frontiers in Bioengineering and Biotechnology, 2017. **5**: p. 24.
